# The effect of eleutherococcus senticosus on metabolism-associated protein expression in 3T3-L1 and C2C12 cells

**DOI:** 10.20463/pan.2020.0016

**Published:** 2020-09-30

**Authors:** Takeshi Hashimoto, Yoko Okada, Atsushi Yamanaka, Natsuhiko Ono, Keisuke Uryu, Isafumi Maru

**Affiliations:** 1 Faculty of Sport and Health Science, Ritsumeikan University, Shiga, Japan; 2 Bizen Chemical Co., Ltd., Akaiwa, Okayama, Japan

**Keywords:** adipocytes, skeletal muscle, exercise, AMPK, mitochondria, lipolysis, lipogenesis

## Abstract

**[Purpose]:**

In vivo studies have demonstrated the ergogenic benefits of eleutherococcus senticosus (ES) supplementation. ES has been observed to enhance endurance capacity, improve cardiovascular function, and alter metabolic functions (e.g., increased fat utilization); however, the exact mechanisms involved remain unknown. We aimed to determine whether ES could effectively induce fat loss and improve muscle metabolic profiles through increases in lipolysis- and lipid metabolism-associated protein expression in 3T3-L1 adipocytes and C2C12 skeletal muscle cells, respectively, to uncover the direct effects of ES on adipocytes and skeletal muscle cells.

**[Methods]:**

Different doses of ES extracts (0.2, 0.5, and 1.0 mg/mL) were added to cells (0.2 ES, 0.5 ES, and 1.0 ES, respectively) for 72 h and compared to the vehicle control (control).

**[Results]:**

The intracellular triacylglycerol (TG) content significantly decreased (p < 0.05 for 0.2 ES, p < 0.01 for 0.5 ES and 1.0 ES) in 3T3-L1 cells. Adipose triglyceride lipase, which is involved in active lipolysis, was significantly higher in the 1.0 ES group than in the control group (p < 0.01) of 3T3-L1 adipocytes. In C2C12 cells, the mitochondrial protein voltage-dependent anion channel (VDAC) was significantly increased in the 1.0 ES group (p < 0.01). Furthermore, we found that 1.0 ES activated both 5' AMP-activated protein kinase (AMPK) and acetyl-CoA carboxylase (ACC) in skeletal muscle cells (p < 0.01).

**[Conclusion]:**

These findings suggest that ES extracts decreased TG content, presumably by increasing lipase in adipocytes and metabolism-associated protein expression as well as mitochondrial biogenesis in muscle cells. These effects may corroborate previous *in vivo* findings regarding the ergogenic effects of ES supplementation.

## INTRODUCTION

In a review article by Goulet and Dionne [1], the authors noted that endurance exercise training enables skeletal muscle to derive a greater proportion of the fuel supply from fat by means of structural and metabolic adaptations [2]. Given that greater fat utilization attenuates the rate of endogenous carbohydrate depletion [2-4], which may potentially increase prolonged exercise performance [4], this physiological adaptation is important for endurance athletes. Some studies using supplementation with *Eleutherococcus senticosus* (ES) have reported an improvement in the ability to obtain energy from aerobic metabolism by increasing oxygen consumption and utilization of fatty acids (FAs) as an energy source, thereby enhancing performance. These benefits might be based on the action of eleutherosides, which can induce various physiological responses and are present in the root of ES (Siberian ginseng) [1].

Kuo et al. (2010), in a randomized controlled study, demonstrated that eight weeks of ES supplementation has ergogenic benefits for recreationally trained males: ES might enhance endurance capacity, improve cardiovascular function, and alter metabolic functions (e.g., increased FA utilization) [5]. However, understanding the exact mechanisms involved require further investigation.

Theoretically, strategies that include exercise training to activate FA oxidation in muscles (i.e., fat utilization) and lipolysis in adipocytes (i.e., fat mobilization) have become preferred methods for metabolic improvement. We previously found that prolonged endurance exercise training significantly decreased epididymal fat mass through increased lipolytic activity, along with an increase in the expression of lipases, such as hormone-sensitive lipase (HSL) and adipose triglyceride lipase (ATGL), in diet-induced obese rats [6]. As for *Panax ginseng*, the effect of ginseng on increased fat metabolism has been well-demonstrated in both animal and human studies (see review article [7]). Regarding skeletal muscle, Sumiyoshi and Kimura demonstrated that ES extract increases beta-oxidation in the skeletal muscle of mice [8]. Hwang et al. (2014) also showed the positive effect of ginseng treatment on increased fat metabolism with endurance training in mice [9].

In addition to its potential to promote FA oxidation in muscle and the significant outcomes associated with increased aerobic metabolism in an in vivo study, it has been reported that *E. henryi* Oliv decreases lipogenesis through alterations of lipogenesis-associated protein expression in 3T3-L1 adipocytes [10]. However, to the best of our knowledge, no study has comprehensively examined the effect of ES on lipid metabolism-associated protein expression in adipocytes or skeletal muscle cells. We hypothesized that ES may have dual effects on adipocytes and skeletal muscle cells, such as elevated fat loss and lipid metabolism, respectively. The aim of this study was to examine whether ES could effectively elicit fat loss and improve muscle metabolic profiles through an increase in lipolysis- and lipid metabolism-associated protein expression in 3T3-L1 adipocytes and C2C12 skeletal muscle cells, respectively, to uncover the direct effects of ES on adipocytes and skeletal muscle cells.

## METHODS

### Cell culture protocol

#### Materials

Extracts of ES (product name: Ukogin; lot. no. UKP-001) were provided by Bizen Chemical Co., Ltd. (Okayama, Japan). The extracts were obtained via ethanol extraction and spray drying. In this study, different doses of ES extracts (0.2, 0.5, and 1.0 mg/mL) dissolved in cell culture media were added to the cells (0.2 ES, 0.5 ES, and 1.0 ES, respectively) and compared to the vehicle control (control).

#### Adipocytes

We cultured 3T3-L1 fibroblasts (American Type Culture Collection, Manassas, USA) in Dulbecco’s modified Eagle medium (DMEM ; 1.0 g glucose/L) supplemented with 10% fetal bovine serum (FBS; Nichirei Bioscience Inc., Messe Düsseldorf, Germany), 100 U/mL penicillin, and 100 μg/mL streptomycin (Nacalai Tesque, Kyoto, Japan ). For differentiation, the cells were grown to confluence and maintained for an additional 48 h, and the medium was replaced with DMEM containing 1 μM dexamethasone, 0.5 mM 3-isobutyl-1-methylxanthine (IBMX), and 5 μg/mL insulin for 48 h. Thereafter, the medium was replaced with DMEM containing 5 μg/mL insulin every 2 days [6,11-13]. On day 5 after differentiation, 3T3-L1 adipocytes were incubated in the vehicle (control) or ES for 72 h.

#### C2C12 myotubes

C2C12 myoblasts (American Type Culture Collection) were grown in DMEM (4.5 g glucose/L) supplemented with 10% FBS, 100 U/mL penicillin, and 100 μg/mL streptomycin (Nacalai Tesque) [13-15]. When the myoblasts were approximately 90%–100% confluent, they were differentiated into myotubes after incubation in DMEM containing 2% horse serum (Thermo Fisher Scientific , Tokyo, Japan). The medium was changed every two days. On day 2 after differentiation, C2C12 myotubes were incubated in ES or the vehicle (control) for 72 h.

### Western blotting analysis

After incubation, the cells were washed with phosphate-buffered saline (PBS) and lysed in RIPA buffer (10 mM Tris-HCl [pH 7.4], 150 mM NaCl, 5 mM EDTA, 0.1% sodium dodecyl sulfate (SDS), 1% Triton, 1% sodium deoxycholate) supplemented with protease inhibitor cocktail (Sigma-Aldrich, St. Louis, USA), phenylmethanesulfonyl fluoride, phosphatase inhibitor cocktail, and PhosSTOP phosphatase inhibitor cocktail (Sigma-Aldrich). The samples were incubated on ice for 1 h and then centrifuged at 15000 × g for 15 min at 4°C. The supernatant was collected, and the protein concentration was determined using a Wako protein assay kit (Fujifilm Wako Pure Chemical Corporation, Osaka, Japan). Equal amounts of protein extracts were separated through 8%–12% SDS-polyacrylamide gel electrophoresis at 40 mA for 1 h and transferred to polyvinylidene fluoride membranes at 60 V for 2 h. After the membranes were blocked for 30 or 60 min at room temperature with Blocking One P (Nacalai Tesque) or skim milk (Fujifilm Wako Pure Chemical Corporation), respectively, they were incubated with primary antibodies against 5’ AMP-activated protein kinase (AMPK) (1:1000; Cell Signaling, Danvers, USA; #2793), phosphorylated-AMPK (Thr172) (1:1000; Cell Signaling; #2535), acetyl-CoA carboxylase (ACC) (1:1000; Cell Signaling; #3662), phosphorylated-ACC (Ser79) (1:1000; Cell Signaling; #3661), ATGL (1:1000; Cell Signaling; #2138), fatty acid synthase (FAS) (1:2000; Cell Signaling; #3180), HSL (1:1000; Cell Signaling; #4107), phosphorylated-HSL (Ser565) (1:1000; Cell Signaling; #4126), carnitine palmitoyltransferase I (CPT1) (1:2000; Alpha Diagnostic, San Antonio, USA; #CPT1M11-A), voltage-dependent anion channel (VDAC) (1:1000; Cell Signaling; #4866), glucose transporter type 4 (GLUT4) (1:2000; Millipore, Burlington, USA; #07-1404), peroxisome proliferatoractivated receptor (PPAR)-γ (1:1000; Santa Cruz; Dallas, USA; #sc-7196), phosphoenolpyruvate carboxykinase (PEPCK) (1:2000; Santa Cruz; #sc-32879), α-Tubulin (1:1500; Sigma-Aldrich; T8203), and glyceraldehyde 3-phosphate dehydrogenase (GAPDH) (1:8000; Sigma-Aldrich; G9545). α-Tubulin and GAPDH were used as loading controls for 3T3-L1 and C2C12, respectively [16]. Immunoreactive proteins were incubated with anti-mouse IgG (1:10,000) or anti-rabbit IgG (1:10,000), horseradish peroxidase (HRP)-linked whole antibody (Sigma-Aldrich) to detect primary antibody binding. After three washes in TBST for 10 min each, chemiluminescence quantification was performed using the Luminata Forte Western HRP substrate (Millipore) followed by detection with the ImageQuant LAS 4000 system (GE Healthcare, Little Chalfont, UK) [13]. The membranes were then treated with the Luminata Forte Western HRP substrate (Millipore) to visualize the bands, and ImageJ software (National Institutes of Health, Bethesda, USA) was used to quantify the band intensities (expression of proteins in 3T3-L1 cells (n = 9 in each group) and C2C12 cells (n = 9 in each group).

### Intercellular triacylglycerol (TG) assay

After ES treatment, 100 μL of lysis buffer (10 mM Tris, pH 7.4, 1 mM EDTA, 0.1% Triton X-100) was added to the cells of each well, and the cells were recovered. Intracellular TG was measured using the Triglyceride E-test Wako (Fujifilm Wako Pure Chemical Corporation) [13].

### Statistical analyses

All data are presented as the mean ± S.E. Data were assessed by one-way analysis of variance (ANOVA) with Dunnett’s test. A value of p <0.05 was considered statistically significant.

## RESULTS AND DISCUSSION

### The addition of ES to mature adipocytes decreases intracellular TG content

**Figure 1.**
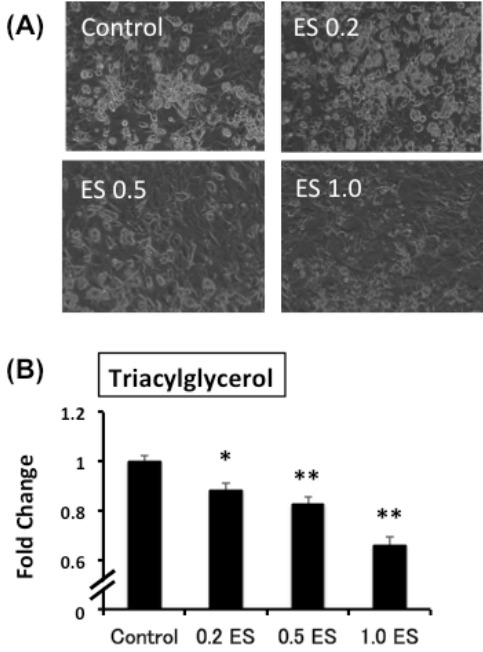
The effect of Eleutherococcus senticosus (ES) on lipid accumulation in mature 3T3-L1 adipocytes. On day 5, mature adipocytes were treated with different concentrations of ES for 72 h (n = 9 for each group). (A) Images of the mature 3T3-L1 adipocytes by optical microscopy as well as (B) TG concentration. *p < 0.05, **p < 0.01 vs. control.

ES was added to mature adipocytes for 72 h, and the intracellular TG content was analyzed. In the ES-treated group, the intracellular TG content was significantly lower than that in the control group (p < 0.05 for 0.2 ES, p < 0.01 0.5 ES and 1.0 ES) (Figure 1).

**Figure 2.**
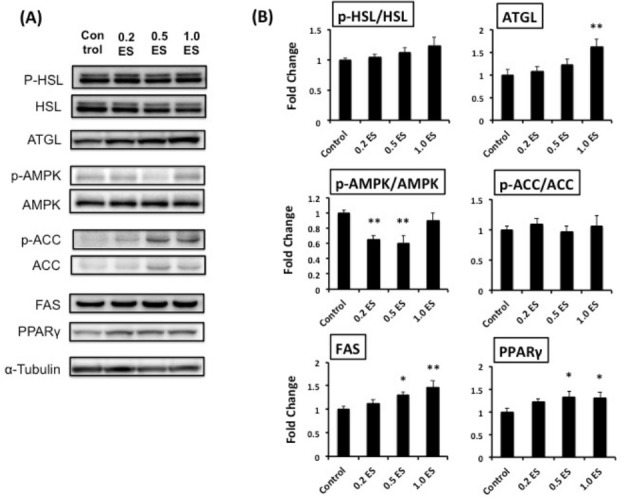
The expression of lipolysis- and lipogenesis-related proteins in mature 3T3-L1 adipocytes. On day 5, mature adipocytes were treated with different concentrations of ES for 72 h (n = 9 for each group). The protein levels are shown relative to control protein levels. *p < 0.05, **p < 0.01 vs. control.

To address the mechanisms underlying the decreased intracellular TG content, we assessed the expression of lipolysis-associated proteins (e.g., lipases), such as ATGL and HSL. We found that ATGL, which is involved in active lipolysis [12], was significantly higher in the 1.0 ES group than in the control (p < 0.01) (Figure 2); this may partially explain the reduced TG content following ES treatment of adipocytes.

We have recently found that fucoxanthinol suppressed fat synthesis by enhancing the phosphorylation of AMPK and ACC, hence decreasing the expression level of FAS [13]. However, ES treatment significantly reduced the phosphorylation of AMPK without any changes in ACC (Figure 2). Furthermore, ES treatment significantly increased the expression of FAS and PPARγ (Figure 2), which may lead to an increase in adipocyte differentiation and adipocyte number. Nonetheless, the significant reduction in TG content by ES treatment is interesting, and the relevant mechanisms should be further elucidated.

### The addition of ES increases metabolism-associated protein expression in differentiated C2C12 skeletal muscle cells

**Figure 3.**
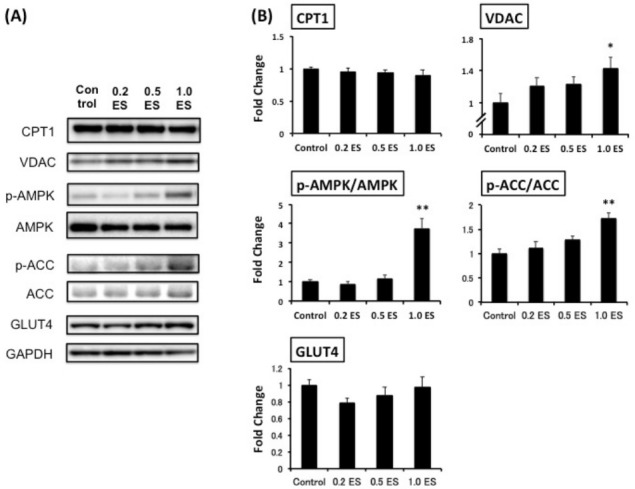
The expression of metabolism-associated proteins in C2C12 myotubes. The protein levels are shown relative to control protein levels. On day 2, muscle cells were treated with different concentrations of ES for 72 h (n = 9 for each group). The protein levels are shown relative to control protein levels. *p < 0.05, **p < 0.01 vs. control.

This study was the first trial to demonstrate the simultaneous effects of ES in decreasing fat in adipocytes and elevating metabolism-associated protein expression in both adipocytes and myotubes (Figure 3). Although CPT1, which is a rate-limiting enzyme of β-oxidation and controls energy metabolism [17], was not affected by ES treatment, and the mitochondrial protein VDAC [18] was significantly increased by 1.0 ES (p < 0.01). Furthermore, we found that 1.0 ES activated both AMPK and ACC in skeletal muscle cells (p < 0.01), suggesting a potential enhancement of fat oxidation. Given that glucose is a key source of energy for skeletal muscle during strenuous exercise [19], and AMPK activation increases Glut4 transcription [20], we also assessed GLUT4 protein expression. However, there was no significant difference in GLUT4 expression. Nevertheless, AMPK activation has been shown to induce GLUT4 translocation to the cell surface irrespective of the increase in GLUT4 expression [21]. In contrast, excessive glucose influx promotes lipid accumulation, while AMPK activation reduces lipogenesis [12]. Further studies are warranted to determine the interaction between AMPK-related glucose and fat metabolism.

## STUDY IMPLICATIONS AND FUTURE PERSPECTIVES

In this study, we investigated for the first time the direct effects of ES extracts on both adipocytes and skeletal muscle cells, such as elevated fat loss and lipid metabolism. The findings suggest that ES extracts, particularly relatively higher doses (e.g., 1.0 mg/mL ES), decrease TG content, presumably by increasing lipases (e.g., ATGL) in adipocytes and metabolism-associated protein expression (e.g., AMPK, ACC) as well as proteins related to mitochondrial biogenesis (e.g., VDAC) in muscle cells. These effects may corroborate the previous *in vivo* findings showing that ES supplementation enhanced endurance capacity, improved cardiovascular function, and altered metabolic functions (e.g., increased fat utilization) [1,5]. Notably, AMPK, a cell energy regulator, plays a major role in the control of glucose and lipid metabolism and hence metabolic disorders, such as diabetes, obesity, and cancer [22,23]. Thus, AMPK has emerged as a therapeutic target for metabolic diseases. Therefore, in obese patients and elderly people, the intake of ES may be useful as an exercise mimetic (i.e., AMPK activator) [24] and may ameliorate metabolic diseases. In addition, mitochondrial dysfunction and subsequent oxidative stress are largely involved in aging, cancer, age-related neurodegenerative conditions, and metabolic syndromes [25]. Exercise represents a viable, non-pharmaceutical therapy with the potential to reverse and enhance the impaired mitochondrial function observed with aging and chronic muscle disuse [26,27]. In this regard, the activation of the exercise mimetic AMPK by ES might enhance VDAC; this can be a potential mechanism to increase the endurance exercise capacity [1,5] as well as a therapeutic approach for aging and metabolic dysfunction [16]. Further *in vivo* studies are warranted to address these issues in the future. In addition, ES treatment significantly increased the expression of FAS and PPARγ, conflicting with the ES-induced decrease in TG content. As mentioned earlier, the mechanisms should be further elucidated, particularly by focusing on the balance between lipolysis and lipogenesis.
